# Using geotagged facial expressions to visualize and characterize different demographic groups’ emotion in theme parks

**DOI:** 10.1038/s41598-024-69555-5

**Published:** 2024-09-09

**Authors:** Xiaoqing Song, Haoze Wu, Wei Jiang, Junjun Zhi, Xinyu Xia, Yi Long, Qin Su

**Affiliations:** 1 School of Geography and Tourism, Anhui Normal University, Wuhu, 241003 China; 2 Key Laboratory of Virtual Geographic Environment, Nanjing Normal University, Nanjing, 210023 China; 3 Wuhu Xingzhan Technology Co., Ltd., Wuhu, China

**Keywords:** Tourists’ emotions, Geotagged facial expression, Theme park, Emotion distribution graph, Psychology, Data acquisition, Data mining, Psychology and behaviour

## Abstract

Tourism is an emotional sphere, and researchers focus on emotions to optimize tourism experiences. Tourism studies on emotions mostly ignore differences in emotions across demographic tourist groups by gender and age, thus limiting the understanding of emotions to the explicit characteristics of tourists’ emotions. On the basis of geotagged facial expressions on social media platforms, this study aims to visualize the emotions of groups in scenic spots and then reveal the variations between groups’ emotions within theme parks. By employing a facial recognition algorithm, an emotion distribution graph was proposed to represent groups’ emotions in detail. Some analytical methods were combined to characterize of the emotion distribution of each group. Through a comprehensive comparison, the results suggest that there are unique characteristics of emotion distribution for each group and considerable variations between them. This study helps researchers achieve a deeper understanding of tourists’ emotional differences and enhances the theorization of emotions. This research also highlights the advantages and significant practical implications of our method framework.

## Introduction

Tourists’ emotions are of great concern in the tourism industry^1^. Both positive and negative emotions are indispensable aspects of tourist activities and have a significant impact on various stages of tourism experiences^2–4^. In the preparation stage, emotions influence tourists’ motivation and their destination choices^5^. During the process of travelling, emotions are strongly related to activity intention and satisfaction^6,7^. Understanding tourists’ emotions is essential for the design of tourism products and the management of tourism destinations. Theme parks are considered a form of leisure activity that attract many tourists^8,9^. Compared with other types of tourism destinations, there is no significant scarcity of theme parks; they can be easily replaced in competitive environments^10^. Many researchers have reported that offering emotional experiences in response to tourists’ growing demand is the key to the sustainable development of the theme park industry^11–13^. As theme parks are artificial tourist destinations, theme park managers should pay more attention to the dynamics of tourists’ emotions.

Measuring and visualizing tourists’ emotions is a challenging task^14^. In theories of psychology, such as the wheel of emotion^15^ and the circumplex model of affect^16^, self-report measures have been widely used to quantify emotion^17,18^. By applying these measures, the emotional states of tourists can be effectively and efficiently captured at different emotion scales^19–21^. Conducting self-report measures is time-consuming, and the data size of measurement results, such as questionnaires and interviews, is limited. In addition, tourists’ memories can be easily distorted after an experience^22^. During the process of completing questionnaires or interviews, misleading information may be generated because of distorted memories. For example, Zhang, Chen and Hsu^23^ unlocked the bias in self-measured emotions by comparing them with emotions extracted from facial expressions. Although some attempts have been made to increase the accuracy of measuring tourists’ emotions^24,25^, both new data resources and new methods need to be utilized to more accurately quantify tourism experiences.

Another consideration in tourist emotion studies is the difference by gender and age. Theme parks can attract tourists with different demographic characteristics, including teens, adults and older people^26–28^. In some scenic spots, tourists may exhibit opposite emotional polarities on the basis of age and gender differences. Exploring emotional differences between demographic groups can provide scientific support for optimizing personal tourism experiences^29,30^. Currently, related studies mainly depend on self-measures and investigate the impact of demographic characteristics on tourists’ emotions at the individual level^20,31^. For instance, Moal-Ulvoas^32^ reported that the emotional needs of teen tourists are quite different from those of older people and revealed significant influencing factors on the emotions of the older people. However, existing tourism studies cannot quantify the complex emotional status of large demographic groups. Furthermore, there is still a lack of available approaches for visualizing the emotions of tourists at different scenic spots within theme parks^14^.

In recent years, social media data, derived from platforms such as Facebook, Twitter, and Sina Weibo, have provided great opportunities for measuring tourists’ emotions^33–35^. Most of these studies have been limited to text analysis by applying natural language processing (NLP)^36,37^. Jiang, Wang, Xiong, Song, Long and Cao^38^ use social media texts to visualize the emotional changes in tourist flows between destinations. With the development of facial expression analysis methods, some studies have begun to examine tourists’ emotions by exploring geotagged facial expressions posted on social media platforms^39–41^. Facial expressions not only contain emotion information but also reflect age and gender with great accuracy. However, tourism emotion research based on facial expressions is still at an early stage, and most existing studies have not distinguished tourist groups^42,43^. To our knowledge, no studies have applied facial expressions to investigate the differences in emotions between tourist groups of distinct demographic characteristics. Given that more effective plans for optimizing tourists’ experiences are needed for theme park management, visualizing and exploring the emotional characteristics of each demographic group will provide more valuable and practical information for optimization strategies.

To fill this gap, geotagged facial expressions on the Sina Weibo platform were applied. This study aims to visualize and thoroughly compare the emotion distributions of different demographic groups. The core research question that guides this study is “What are the differences between demographic groups’ emotions in theme parks?”. A facial recognition algorithm was introduced to explore tourists’ emotions in the Shanghai Disney Resort, China. Specifically, this study first applied FaceReader to obtain demographic information and the emotion status of each facial expression; then, an emotion distribution graph was proposed and constructed to visualize each group’s emotions in different scenic spots; finally, analysis methods were combined to characterize the emotion distribution and reveal variations between groups. The results of our study are not only critical for increasing the attractiveness of tourism destinations for different groups but they also provide new insights into the understanding of the relationship between tourists’ emotions and destination management.

## Related works

### Emotion measure

Emotion is a unique characteristic that distinguishes humans from other creatures^44,45^. The temporal, spatial and social attributes of emotion make it ubiquitous in people’s daily lives. According to Darwin’s theory^46^, emotion is a complex psychological response that is related to neural structures. In addition, some researchers have postulated emotions as bodily and mental reactions based on cognitive appraisals^47,48^. Currently, theoretical approaches, such as the circumplex model of affect^16^ and the wheel of emotion^15^, have been proposed to develop the spectrum of emotion categories. The circumplex model of affect applies two axes, valence and arousal, to classify 28 emotion types^16^. In addition, the wheel of emotion involves eight types of emotion and assumes that all psychological responses can be decomposed into these basic emotions^15^. Although there is no agreement on emotion classification, most scholars agree that emotions can be accurately measured via different methods^25,49,50^.

As a typical and traditional measure, The use of self-reported measures is a typical and traditional approach that includes questionnaires and interviews requiring people to describe their emotions^51^. Questionnaires and interviews record detailed information related to individuals’ emotions. Owing to the emotion expression tendency and the false memory paradigm, self-reported measures may overestimate some positive emotions and mislead recognition of human emotions^52^. Several studies have noted bias in emotions on the basis of these measures^53^. With the development of sensors, skin conductance levels, body temperature and facial expressions that are strongly related to people’s emotions can be collected^54^; these data resources have been proven to be reliable for quantifying emotions^55,56^. For instance, Horn et al.^57^ investigated the changing pattern of emotional responses from depression patients by analyzing their skin conductance levels. Li et al.^58^ revealed the usefulness and suitability of skin conductance levels and other sensor data in exploring tourists’ emotions; they claimed that these data have potential value for future work.

In recent years, owing to the application of deep convolutional neural networks^59^, emotion recognition technology based on facial expressions has made more significant and substantial advancements than other emotion measures^60–62^. Based on Darwin’s expression reflexology^46^ and the corresponding basic emotion theory^63^, most existing methods, such as Face++ and FaceReader, distinguish six basic emotions (anger, disgust, surprise, sadness, fear and happiness) for each facial expression^64^. A large body of valuable findings has shown the effectiveness of these facial expression recognition technologies^65,66^. Recent efforts such as that of Li et al.^67^ have used Face++ to explore facial expressions in geotagged Flickr photos and map the global spatial distributions of some basic emotions. Zhang et al.^23^ focused on the emotional responses of Hong Kong residents to mainland tourists and compared the emotions extracted from facial expressions and interviews. Based on facial expression analysis, Weismayer^68^ monitored the emotional responses of participants watching a tourism destination commercial and then link edemotional responses with specific sequences of the advertisement. As one of the most advanced technologies in application, facial recognition can be considered a measure comparable with self-reported measures.

### Tourist emotion research

Tourist emotion has been an important subfield in tourism research^69,70^. Emotion can influence tourists’ attitudes towards the quality of tourism services, their satisfaction and their behavioral intentions^38,71,72^. According to the theory of Jin et al.^73^, tourists expect not only to receive professional services but also to have emotional experiences. Providing tourists with emotional experiences is essential for maintaining a competitive advantage in the tourism industry^17,74,75^. More attention has been given to tourists’ emotions in the past ten years, and some scholars have suggested that tourists’ emotions will become a leading topic in the future^76–78^. Specifically, emotion-related studies in tourism have focused on (1) the impact of tourists’ emotions on loyalty^79,80^ and destination selection^1,81^; (2) the dimensional structure and the dynamic changes of tourist emotion^69,82,83^; and (3) the influencing factors of tourists’ emotions^84^. A large body of valuable findings in tourism research has demonstrated that emotion is an important indicator in tourist experience management^85,86^.

The concept of a modern theme park originated in Disneyland, which was built in 1955 in Anaheim, California in the U.S. Theme parks are comprehensive leisure and entertainment sites that are artificial and based on specific themes^87–89^. As an important type of tourism destination, theme parks focus on their emotional appeal for tourists and pay much attention to the optimization of emotional experiences^26,53^. Related research on theme parks can be classified into macro and micro levels^90^. Most macrolevel studies have investigated the impacts of transportation^69^, holidays^70^ and cutting edge technology^73^ on the development of modern theme parks. At the micro levels, recent scholarly efforts focused on the various factors influencing tourists’ emotions and behaviors. For instance, an empirical study based on cognitive appraisal theory showed that the degree of goal realization and novelty are strongly related to tourists’ delight and satisfaction^17^. Many studies have suggested that emotional status is vital for leading theme park tourists’ behavior and intentions^74^. However, to the best of our knowledge, no further effort has been made to investigate the differences in emotions across demographic groups.

### Tourism research based on social media data

With the development of mobile communication technology, social media services, such as Twitter, Facebook and Sina Weibo, have been widely used by both tourists and marketers in modern society^89,91,92^. For tourists, social media platforms are an available means of searching for tourism information, posting about travel experiences and communicating with other tourists. Marketers are employed by tourism brands to effectively promote them and improve their competitive advantages. Currently, exploring the impact of social media on tourism development has become a hot topic in the tourism academic community^93–95^. Usui et al.^96^ took Ōkunoshima Island in Japan as a case study; they revealed the power of social media by investigating the relationship between the spread of online videos and the increase in the number of tourists. Social media has helped form a new marketing mode for the tourism industry and plays an integral role in communication between tourists^97,98^.

Many scholars have found social media data to be a reliable data resource for analyzing tourists’ opinions and activities^1,82^. According to the theory of Goodchild^76^, each tourist can be considered a “social sensor” that produces large amounts of user-generated content (UGC), such as texts, images and videos. By applying a topic model^71^, several studies have discussed the topics in which tourists are interested and the formation mechanism of these topics^89,99,100^. In addition, some tourism studies have focused on revealing spatiotemporal travel patterns by combining many approaches, such as trend decomposition^101^ and predictive analytics^79^. For instance, Mou et al.^72^ utilized network analysis to characterize tourist flows between different destinations by applying travel diaries on a social media platform.

Social media data reflect a variety of detailed information about tourists’ emotions and have been applied to emotion analysis in tourism research^102–104^. With the advancement of natural language processing, it is more feasible to quantify tourists’ emotions based on social media texts. For instance, Park et al.^84^ mined the positive and negative emotional strengths from Twitter texts and then constructed the relationship between tourists’ emotions and places at Disneyland. In the last decade, many facial expressions in photos have been posted on social media platforms and can be collected from Twitter, Facebook and Sina Weibo. Several scholars have utilized tourists’ facial expressions in photos to measure their emotions via facial recognition technology^105^. Compared with text, facial expressions are a more accurate and effective data resource for quantifying emotions^85^. People worldwide the world have similar basic facial expressions^67^. Existing evidence has suggested that the emotion extracted from facial expressions is consistent across cultures, nations, genders and ages^86^. Theme parks attract tourists with different demographic characteristics and cultural backgrounds. Therefore, facial expressions are suitable for analyzing tourists’ emotions in theme parks.

## Methods

This section includes the following subsections: data collection and preprocessing, facial recognition, emotion distribution graph construction, and analysis methods. The data collection and preprocessing section explains how the dataset was collected and cleaned. The facial recognition section represents how human emotion, age and gender were quantified. The emotion distribution graph construction illustrates the process of visualizing each group’s emotions in different scenic spots. The analysis method section describes the approaches that were applied to quantify each emotion distribution.

### Data collection and preprocessing

**Figure 1:**
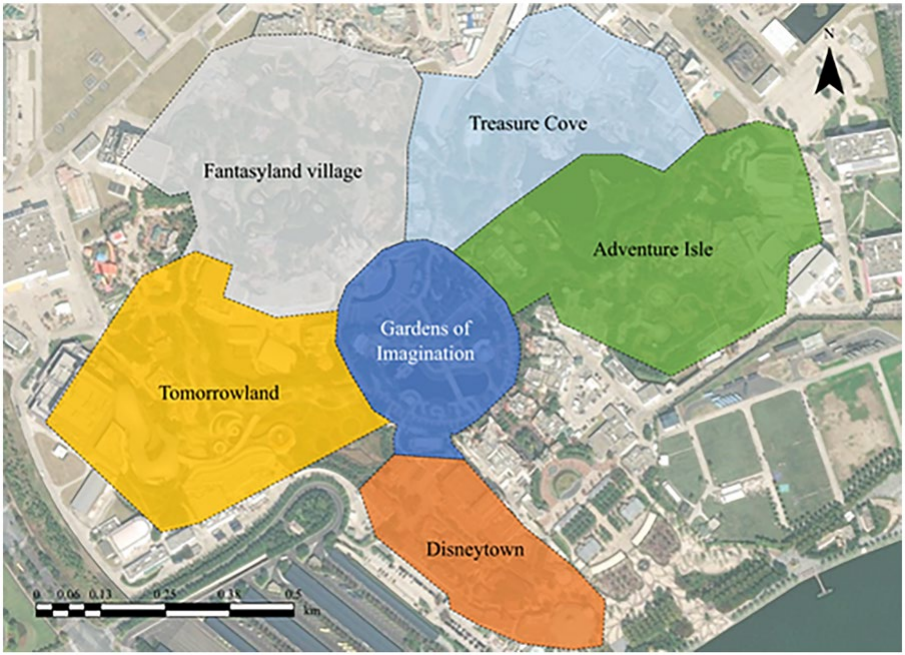
The distribution of theme lands in Shanghai Disney Resort. The spatial distributions of 6 theme lands, with 6 different colors, are adjacent to each other. Figure was created using ArcGIS 10.8.2 (https://www.esri.com/en-us/home).

This study collected geotagged microblogs from Sina Weibo, one of the largest social media services in China. Sina Weibo is the Chinese version of Twitter and provides an application programming interface (API) for collecting microblogs. By applying the Sina API, we collected 227,239 geotagged microblogs within the Shanghai Disney Resort from January 2019 to December 2020. Many scholars have noted that there is a certain amount of noise in social media data^106–108^. Based on the preprocessing method proposed by previous studies^38,109^, the noise was filtered out, and 42,988 microblogs were retained. After preprocessing, 148,132 geotagged facial expressions were identified from the images in the microblogs. Although there is bias in tourists’ emotions based on online data, many existing studies have shown that online facial expressions can reflect tourists’ emotion effectively^14,53,110,111^. The Shanghai Disney Resort was built in 2016 and features 6 theme lands: Treasure Cove, Fantasyland, Tomorrowland, Gardens of Imagination, Adventure Isle, and Disneytown. The spatial distribution of these theme lands is shown in Fig. 1.

Based on the context of the collected images, the images can be categorized into people-related and scene-related images. Most people-related images are posted to record the emotions of the publisher; the main contexts of these images involve the publishers and their friends. The scene-related images reflect the environment within the theme park. The spatial density of tourists within Shanghai Disney Resort is high, especially on holidays. Both image types contain many facial expressions of other people who are included in the background. By analyzing the image contexts, the facial expressions of other people constitute the major proportion of all facial expressions.

### Facial recognition

The FaceReader Cognitive Algorithm was applied in this study to recognize the emotions and demographic attributes of facial expressions. FaceReader has proven to be an effective method of estimating facial emotions with a high degree of accuracy by many previous studies^112,113^. In addition, this algorithm can recognize facial expressions with small areas or low pixels in the images. Therefore, most facial expressions of tourists happen to be included in the background can be translated into emotions successfully. The reports of FaceReader present measures of emotion on two scales. The algorithm can classify facial expressions into seven dimensions: happiness, sadness, disgust, anger, surprise, fear and neutral. In addition, the valence and arousal value of each facial expression can be quantified. In the circumplex model of affect, valence is a measure of pleasure, and arousal refers to excitement quantification. Seven basic dimensions are not sufficient for describing the complex emotions of tourists, and it is difficult to distinguish slight differences between tourists. Therefore, the valence and arousal values were applied in this study.

Recognizing the gender and age associated with each facial expression is the premise of tourist group classification. By applying the FaceReader Cognitive Algorithm, 42,595 facial expressions were recognized as male. After the gender of each facial expression was obtained, the corresponding age was identified. Based on the theories of Huang et al.^53^ and the results of age recognition, we first categorized tourists into three age groups: teenagers (age < 20), adults (age 20–50) and older people (age > 50). The three groups were further categorized into 6 groups according to sex: older male (OM), older female (OF), adult male (AM), adult female (AF), teen male (TM) and teen female (TF). The statistical results of the tourist groups are shown in Table 1.

**Table 1:** The statistical results of the tourist groups.

	OM	OF	AM	AF	TM	TF
Total	2192	3270	24,277	64,539	3312	7168

After the facial recognition results were stored, all the raw images in our dataset were cleared. Images may contain private information about tourists. To protect personal information and follow security guidelines, no images were retained or directly applied for further analysis.

### Emotion distribution graph construction

**Figure 2:**
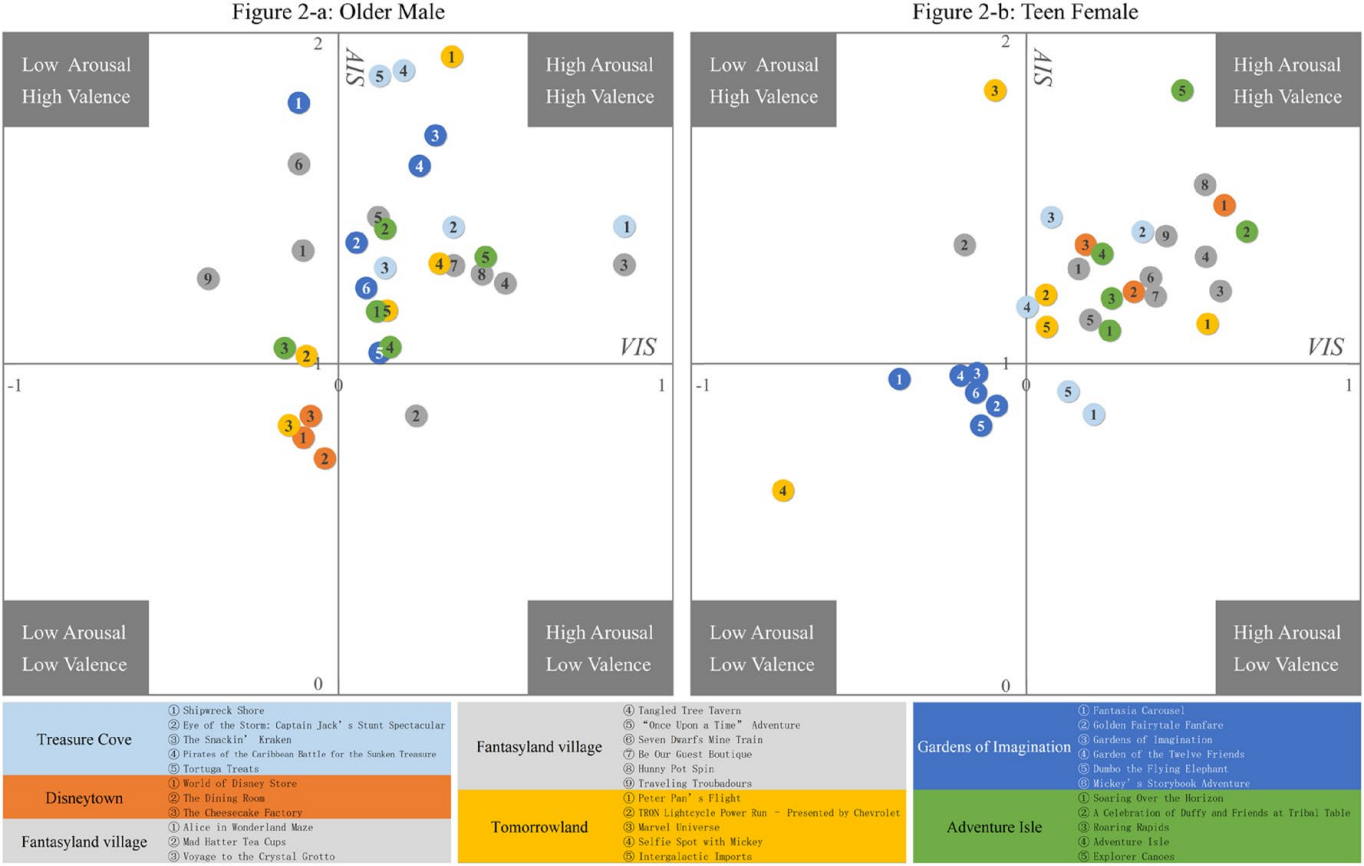
Examples of emotion distribution graphs. The emotion distribution graph, with color of each point representing the theme land of the scenic spot, with the number inside each point representing the serial number for the scenic spot. Figures were created using Python 3.8.10 (https://www.python.org/).

Based on the circumplex model of affect, an emotion distribution graph was proposed to visualize the emotions of each demographic group in different scenic spots. The graphs for the OM and TF groups are shown in Fig. 2. The graph contains the axis of the valence intensity index for the scenic spot ($VIS$) and the arousal intensity index for the scenic spot ($AIS$). Each graph corresponds to a demographic group. There are points in the graph for evaluating the target group’s emotions in different scenic spots. Each evaluation point corresponds to a scenic spot. The numerical symbols of the evaluation points in the graph represent the serial numbers of the scenic spots. The colors of the points indicate the theme lands of the corresponding scenic spots. For instance, there are 9 serial numbers inside grey points in Fig. 2a, which means that there are 9 scenic spots in the theme land of Fantasyland village in total. There are four quadrants in the graph. The first quadrant indicates positive emotional tendencies and high arousal levels. In contrast, the third quadrant presented negative emotions and low arousal levels.

Each evaluation point corresponds to a pair of emotion coordinates that contain $VIS$ and $AIS$. Calculating emotion coordinates was the key to mapping these points graphically. Based on the valence and arousal value of each geotagged facial expression, the emotion coordinate of the evaluation point can be calculated as follows:

1 $${VIS}_{i}^{g}=\frac{{VS}_{i}^{g}}{{N}_{i}^{g}},$$

where ${N}_{i}^{g}$ refers to the sum of the facial expressions of group $g$ within scenic spot *i*. The locations of facial expressions are represented as the geographical coordinates of their corresponding images. The total number of geotagged facial expressions posted within the spatial extent of scenic spot *i* was calculated as ${N}_{i}^{g}$. ${VS}_{i}^{g}$ is the sum of the valence values of group $g$ in scenic spot *i* and can be calculated as the total value of the valence of those facial expressions. The $AIS$ for group $g$ in scenic spot *i* is defined as follows:

2 $${AIS}_{i}^{g}=\frac{{AS}_{i}^{g}}{{N}_{i}^{g}},$$

where ${AS}_{i}^{g}$ can be calculated as the total valence value of the facial expressions of group $g$ within scenic spot *i.* After calculating $VIS$ and $AIS$, the emotion distribution can be visualized in the graph and applied for further analysis.

### The analysis method of emotion distribution

In this part, the standard deviational ellipse (SDE) and intensity indices were combined to quantify the overall characteristics of the emotion distribution. The density-based spatial clustering of applications with noise (DBSCAN) was applied to detect the aggregation phenomenon within the emotion distribution. SDE was employed to quantify the direction and central tendency of the emotion distribution. Sixty-eight percent of the points in the graph will be contained in the ellipse. The mean center, azimuth and standard distance are the core indices in the SDE. The mean center is a widely used index to measure central tendency and can be calculated as follows:

3 $${SDE}_{V}=\sqrt{\frac{\sum_{i=1}^{n}{\left({VS}_{i}^{g}-\overline{{VS }^{g}}\right)}^{2}}{n}},$$

4 $${SDE}_{A}=\sqrt{\frac{\sum_{i=1}^{n}{\left({AS}_{i}^{g}-\overline{{AS }^{g}}\right)}^{2}}{n}},$$

where *n* refers to the number of scenic spots. The azimuth *θ* was calculated as follows:

5 $${\text{tan}}\theta =\frac{A+B}{C},$$

6 $$A = \mathop \sum \limits_{i = 1}^{n} \widetilde{VS}_{i}^{g2} - \mathop \sum \limits_{i = 1}^{n} \widetilde{AS}_{i}^{g2} ,$$

7 $$B = \sqrt {\left( {\mathop \sum \limits_{i = 1}^{n} \widetilde{VS}_{i}^{g2} - \mathop \sum \limits_{i = 1}^{n} \widetilde{AS}_{i}^{g2} } \right)^{2} + 4\left( {\mathop \sum \limits_{i = 1}^{n} \widetilde{VS}_{i}^{g} \widetilde{AS}_{i}^{g} } \right)^{2} } ,$$

8 $$C=2\sum_{i=1}^{n}{\widetilde{VS}}_{i}^{g}{\widetilde{AS}}_{i}^{g}.$$

The standard distances in the $VIS$ and $AIS$ axes were calculated via Eqs. (9) and (10).

9 $${\sigma }_{V}=\sqrt{\frac{2\sum_{i=1}^{n}{\left({\widetilde{VS}}_{i}^{g}{\text{cos}}\theta -{\widetilde{AS}}_{i}^{g}{\text{sin}}\theta \right)}^{2}}{n}},$$

10 $${\sigma }_{A}=\sqrt{\frac{2\sum_{i=1}^{n}{\left({\widetilde{VS}}_{i}^{g}{\text{sin}}\theta +{\widetilde{AS}}_{i}^{g}{\text{cos}}\theta \right)}^{2}}{n}}.$$

DBSCAN is a clustering method based on density and was introduced to detect the aggregation phenomenon in the emotion distribution graph. The X and Y coordinates of each point in the graph refers to the valence and arousal values, respectively. Therefore, the distance between two points in the graph can be considered to represent the similarity between the emotions of scenic spots. The closer the two points, the more similar their emotions. By applying DBSCAN, we can identify the clusters of scenic spots whose emotions are close to each other. Each cluster corresponds to an aggregation phenomenon. Tourists have similar emotional experiences within scenic spots in the same cluster. In other words, these scenic spots in the same cluster can motivate similar emotions, resulting in an aggregation phenomenon. Eps and MinPts are two core parameters in this algorithm. Eps is the search radius and MinPts is the minimum number of points required to form a cluster. Unlike other clustering methods, DBSCAN does not need the number of clusters as a priori knowledge and can recognize arbitrarily shaped clusters. Based on the experience of previous studies^114^, Eps was set as 0.11, and MinPts was set as 4.

The valence intensity index for theme land ($VIT$) and the arousal intensity index for theme land ($AIT$) were proposed to evaluate the comprehensive emotions of the target group in different theme lands. Specifically, the $VIT$ for the TF group in theme land *j* can be calculated as follows:

11 $${VIT}_{j}^{g}=\frac{\sum_{l=1}^{m}{VS}_{l}^{g}}{m},$$

where $m$ refers to the total number of scenic spots in theme land $j$. $AIT$ was defined as follows:

12 $${AIT}_{j}^{g}=\frac{\sum_{l=1}^{m}{AS}_{l}^{g}}{m}.$$

## Results

### Emotion distribution analysis

**Figure 3:**
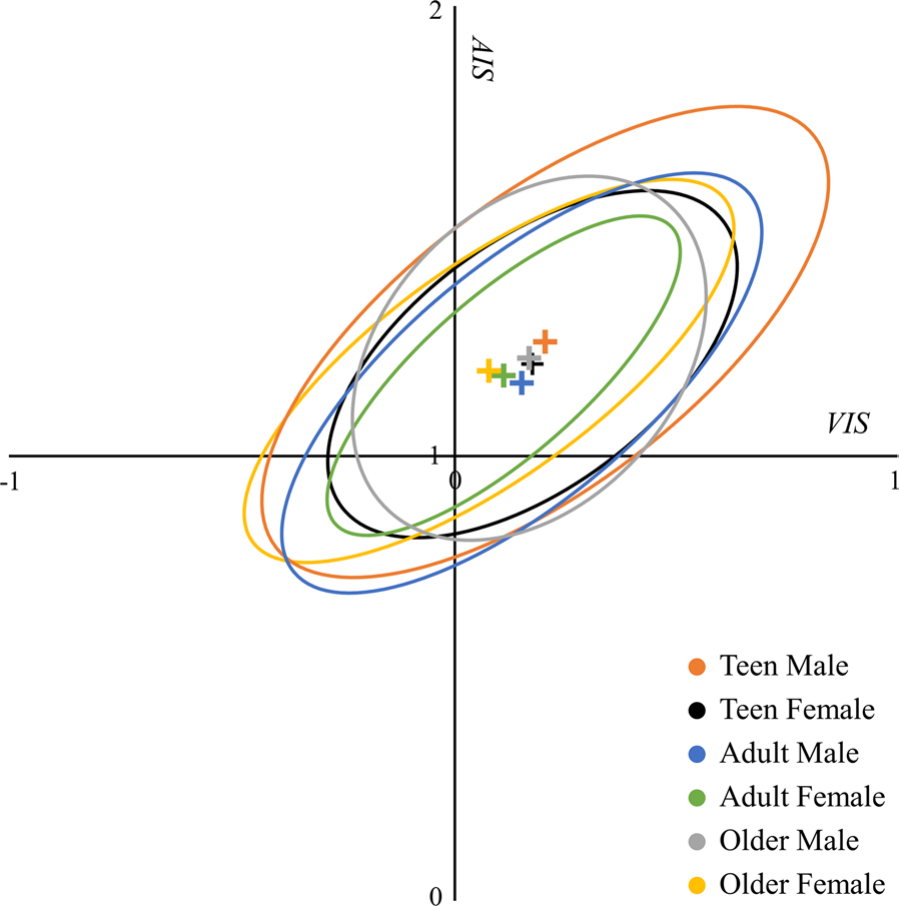
The ellipses of the graphs. The ellipses of emotion distributions, visualizing and quantifying the trend differences between demographic groups. Figures were created using Python 3.8.10 (https://www.python.org/).

The results of the SDE for each graph are shown in Table 2, and all ellipses with mean centers are shown in Fig. 3. To validate whether the differences across ellipses were statistically significant, Friedman tests were applied. All p values of the Friedman tests were lower than 0.01, which indicates that the differences were significant. ${SDE}_{V}$ and ${SDE}_{A}$ can describe the location of the ellipse in the graph. All ${SDE}_{V}$ values were positive and all ${SDE}_{A}$ values were greater than 1.0; this finding indicates that all the mean centers of ellipses are in the first quadrant. There are differences between locations of the ellipses. ${SDE}_{V}$ ranges from 0.075897 to 0.205197 and ${SDE}_{A}$ ranges from 1.158367 to 1.268219. Among all the demographic groups, the TMs have the highest values of the mean center. The azimuth ($\theta$) is the direction of the ellipse, and all the graphs are similar to each other. The directions of all ellipses were from the first quadrant to the third quadrant. Specifically, the largest azimuth occurred in OFs (54.1), followed by those in TFs (53.1), TMs (52.5), AMs (50.3), AFs (48.9), and OMs (42.1). The standard distance determines the coverage area of the ellipse. From Table 2 and Fig. 3, TMs have the highest standard distance in both the $VIS$ and $AIS$ axes; a high standard distance corresponds to a large emotion distribution area in the graph. Figure 3 shows that the ellipse of AFs has the smallest area.

**Table 2:** The results of the SDE for each graph.

	TM	TF	AM	AF	OM	OF
${SDE}_{V}$	0.205197	0.170726	0.138065	0.106733	0.166782	0.075897
${SDE}_{A}$	1.268219	1.211051	1.158367	1.184262	1.228382	1.184016
${\sigma }_{V}$	0.323862	0.273387	0.261099	0.171486	0.323538	0.233309
${\sigma }_{A}$	0.756172	0.528915	0.653621	0.493562	0.452164	0.641399
$\theta$	52.501387	53.055161	50.268647	48.865419	42.142231	54.117766

**Figure 4:**
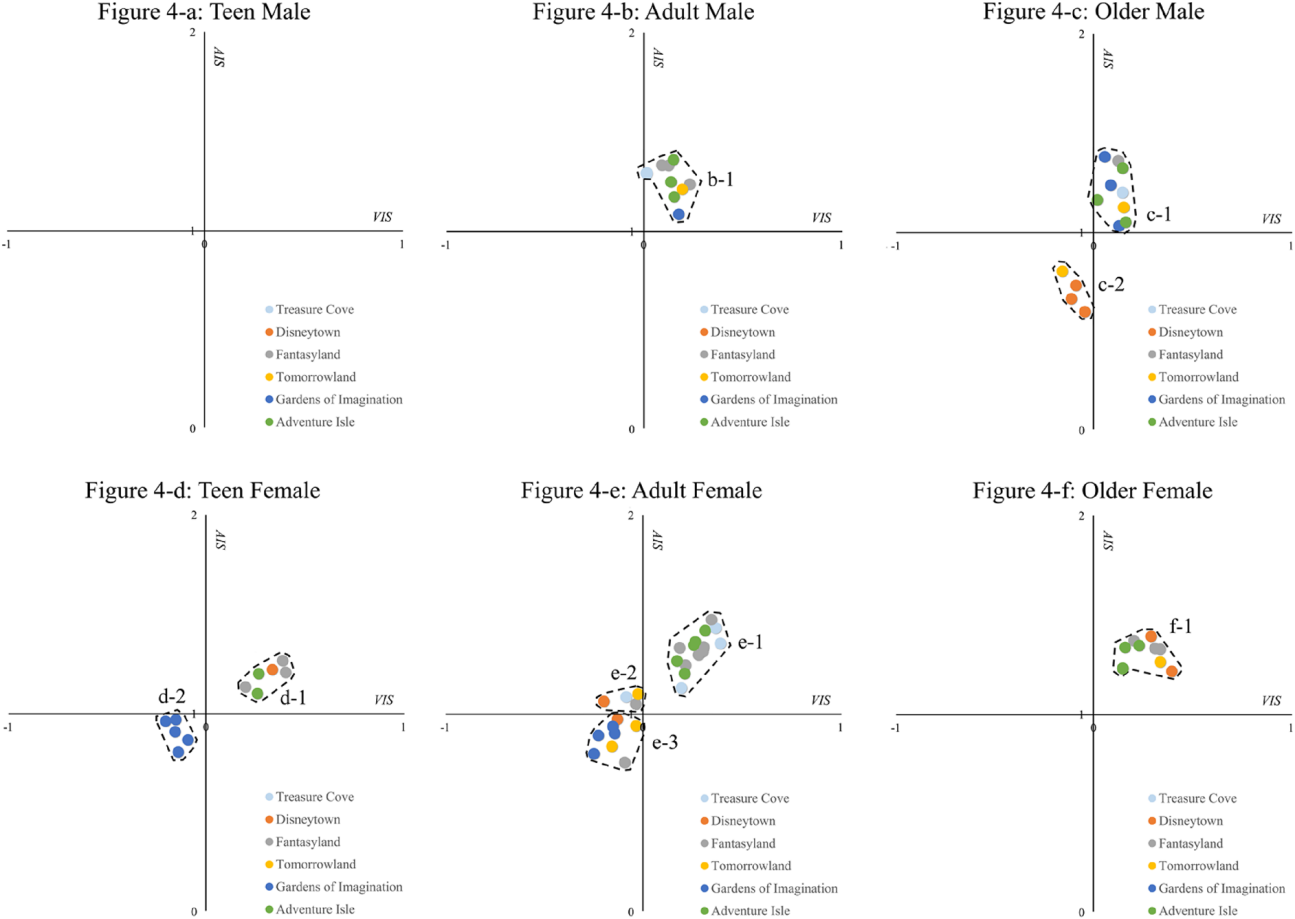
The cluster in each graph. The clusters of emotion distributions, recognizing points that have similar emotions. Figures were created using Python 3.8.10 (https://www.python.org/).

The results of DBSCAN are shown in Fig. 4 and Table 3. There are obviously different numbers and shapes of aggregation phenomena (clusters) across the emotion distribution graphs. TMs have no emotion cluster; this means that the TM emotions in each scenic spot vary significantly and cannot form a cluster. Both AMs and OMs have one cluster in the first quadrant. The cluster indicates that the group’s emotions in some scenic spots were similar to each other. Both the TFs and OMs have two clusters. Compared with the other groups, AFs have the most clusters. The e-1 and the e-2 clusters are located in the first and second quadrants, respectively. The e-3 cluster is in the third quadrant, which demonstrates that some scenic spots evoke a similar negative valence and similar low arousal of AFs.

**Table 3:** Description of the clusters in each graph.

Group	Number of clusters	Cluster name	Number of points in cluster
TM	0	None	None
AM	1	b-1	9
OM	2	c-1	9
		c-2	4
TF	2	d-1	6
		d-2	5
AF	3	e-1	14
		e-2	4
		e-3	8
OF	1	f-1	9

$VIT$ and $AIT$ can evaluate the trend of groups’ emotions in different theme lands. The $VIT$ and $AIT$ for each group are shown in Table 4 and can be summarized as follows:

- For TMs, the highest value of $VIT$ is in Disneytown, and the lowest value is in Adventure Isle. The highest value of $AIT$ is in the Gardens of Imagination, and the lowest value is in Adventure Isle.
- For TFs, both the highest values of $VIT$ and $AIT$ are in Adventure Isle. Both the lowest values of $VIT$ and $AIT$ are in the Gardens of Imagination.
- For AMs, the highest value of $VIT$ is in the Gardens of Imagination, and the highest value of $AIT$ is in Adventure Isle. Both the lowest values of $VIT$ and $AIT$ are in Disneytown.
- For AFs, the highest value of $VIT$ is in Treasure Cove, and the highest value of $AIT$ is in Adventure Isle. Both the lowest values of $VIT$ and $AIT$ are in the Gardens of Imagination.
- For OMs, the highest value of $VIT$ is in Treasure Cove, and the highest value of $AIT$ is in the Gardens of Imagination. Both the lowest values of $VIT$ and $AIT$ are in Adventure Isle.
- For OFs, the highest value of $VIT$ is in Disneytown, and the highest value of $AIT$ is in Adventure Isle. Both the lowest values of $VIT$ and $AIT$ are in the Gardens of Imagination.

**Table 4:** The $VIT$ and $AIT$ of each group.

	TM		TF		AM		AF		OM		OF	
	$VIT$	$AIT$	$VIT$	$AIT$	$VIT$	$AIT$	$VIT$	$AIT$	$VIT$	$AIT$	$VIT$	$AIT$
Treasure Cove	− 0.27	0.94	0.16	1.16	0.29	1.33	0.35	1.28	0.34	1.42	0.25	1.32
Disneytown	0.64	1.42	0.38	1.36	− 0.33	0.72	− 0.08	1.15	− 0.08	0.67	0.33	1.24
Fantasyland	0.38	1.34	0.34	1.31	0.06	1.14	0.23	1.23	0.21	1.23	0.12	1.19
Tomorrowland	0.33	1.35	− 0.03	1.18	− 0.08	0.96	0.06	1.26	0.11	1.19	0.26	1.32
Gardens of Imagination	0.45	1.74	− 0.19	0.91	0.43	1.27	− 0.26	0.89	0.16	1.42	− 0.52	0.68
Adventure Isle	− 0.30	0.73	0.39	1.38	0.27	1.34	0.24	1.31	0.12	1.18	0.20	1.35

For some groups, the motivation effects of theme land on valence and arousal differ. For example, Disneytown evokes the strongest positive emotions of TMs, but does not obtain the highest state of arousal.

### Differences between emotion distributions

To further illustrate the differences between emotion distributions, the graphs with scenic spot information were visualized and compared. The Friedman tests were applied to validate whether the differences were significant.

#### Teen males and teen females

**Figure 5:**
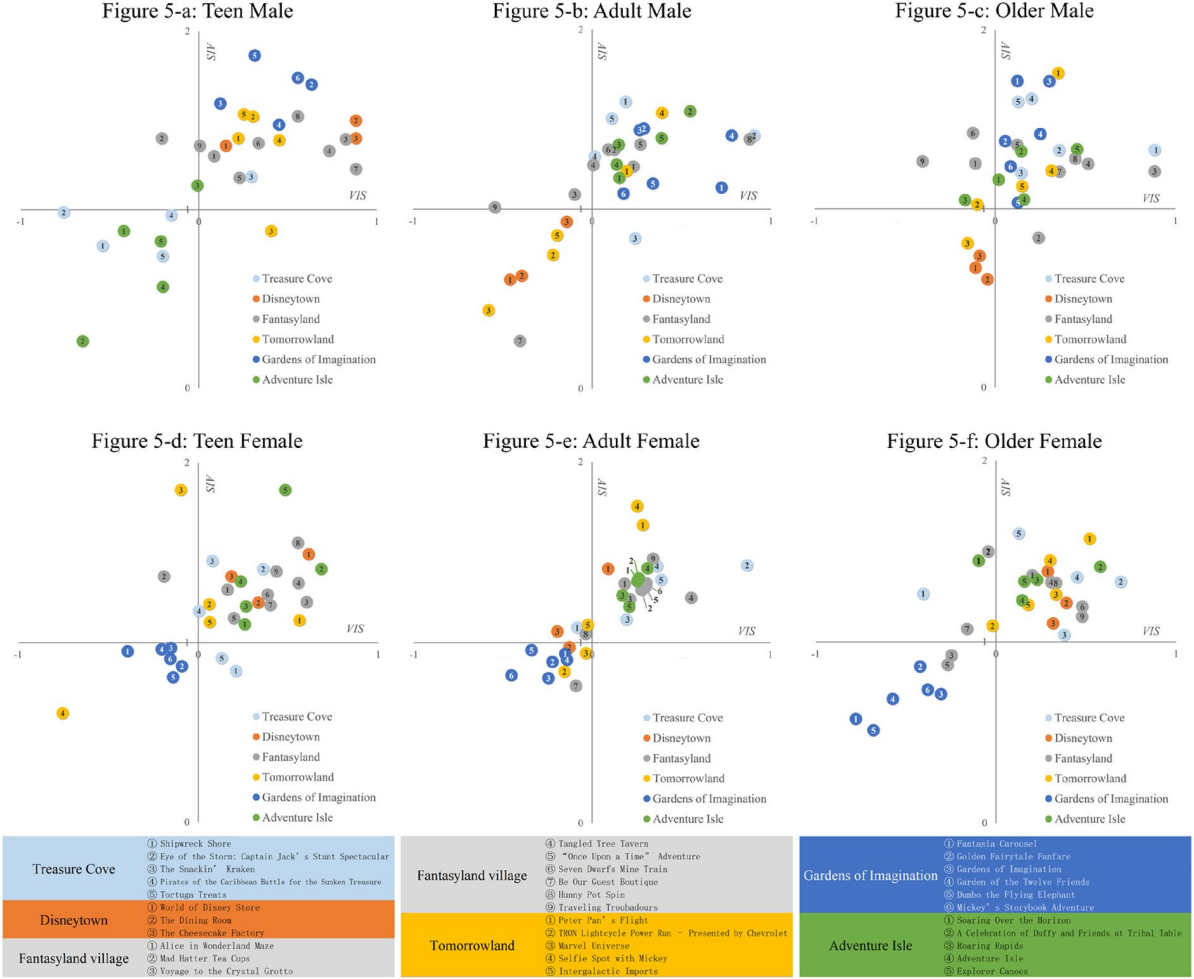
The emotion distribution graph of each group. The emotion distribution graph visualizing and quantifying the differences in emotions across demographic groups in detail. Figures were created using Python 3.8.10 (https://www.python.org/).

The difference between TMs and TFs was statistically significant (p value < 0.01). By comparing Fig. 5a,d, we can identify the differences between the TM and TF emotions in Treasure Cove (in light blue) and Adventure Isle (in green). Most light blue and green TF points are in the first quadrant. The points in the first quadrant have high $VIS$ and $AIS$ values. There are adventure activities in these two theme lands. Through text analysis, we find that adventure activities significantly evoke the emotions of the TFs. For instance, a TF tourist posted, “Roaring Rapids is very exciting ah, much more fun than Fangte”. Most light blue and green TF points are in the third quadrant; these points indicate that the TMs have negative emotions and do not feel much excitement in most scenic spots. Compared with the TFs, the TMs show less pleasure and excitement in Treasure Cove and Adventure Isle.

There are considerable variations between the TMs and TFs in the Gardens of Imagination (in dark blue). The dark blue TM points are mostly in the upper right of the same color TF points. The TM tourists feel more pleasure and excitement than the TFs in the Gardens of Imagination. The Gardens of Imagination contains a carousel and an open garden. In general, the Gardens of Imagination was designed to attract female tourists. However, the Gardens of Imagination can attract TMs as well. Based on the text analysis, we find that the TMs show much pleasure and excitement. For example, a TM tourist posted, “This castle is my Disney dream. The moment I saw it, I felt like I had earned it. As the saying goes, ‘Everyone has a princess dream in their heart’”.

In Disneytown (in orange), the orange TM and TF points are all in the first quadrant. This means that both the TMs and TFs are interested in the scenic spots in Disneytown. There are slight differences between the TMs and TFs. The TMs are more interested in the dining rooms (Cheesecake Factory). However, the TFs are more interested in shopping (Disney Store). Similar findings can also be drawn from social media texts. For example, a TM tourist posted, “It is just town fun; you can eat Disney cakes without a ticket. You can bring your girlfriend to buy her the little dress that she truly wants”.

#### Teen males, adult males and older males

The differences between TMs, AMs and OMs are statistically significant (p value < 0.01). A comparison of Fig. 5a–c reveals that the dark blue points of the TMs, AMs and OMs are all in the first quadrant. All the male tourists were interested in the Gardens of Imagination. All orange AM and OM points are in the third quadrant. Compared with the TMs, both the AMs and OMs displayed less pleasure and excitement in Disneytown (in orange). The texts revealed that some AMs and OMs complained about the high price of the food and other goods. For example, an AM tourist posted, “Why so expensive? Next time, I will bring my own hot rice”.

A special phenomenon regarding the AMs is revealed in Fig. 5b. The five yellow AM points nearly form a line. Tomorrowland (in yellow) is designed to exhibit the primary imagery of Disney movies. The release dates of the primary imagery are shown in Table 5. By analyzing the relationship between the release date and the $VIS$ of the AMs, we find that the AMs are more interested in primary images that were released earlier. The texts of the AMs support our findings. For example, “I miss watching Miss Rice and Donald Duck on TV as a kid”.

**Table 5:** The release dates of the primary imagery of the Disney movies.

Scenic spot	Primary imagery of Disney movies	Release date	$VIS$ of AMs
Selfie spot with mickey	Mickey	1980	0.3898
Peter Pan’s Flight	Peter Pan	1991	0.1933
Intergalactic imports	Star Wars	2000	− 0.096
TRON lightcycle power run	Genesis	2011	− 0.319
Marvel universe	Marvel Hero	2008–now	− 0.578

#### Teen females, adult females and older females

The differences between the TFs, AFs and OFs were statistically significant (p value < 0.01). As shown in Fig. 5f, all the dark blue OF points are in the third quadrant. Compared with the TFs and AFs, the OFs have clear negative emotions and a low level of arousal in the Gardens of Imagination (in dark blue). The OFs thought that the factors in the Gardens of Imagination were naïve, and they were not attracted by this theme. For example, an OF tourist posted, “This place is full of little girl stuff”. Like those of the TFs, all orange points of the OFs are in the first quadrant. The OFs are very interested in Disneytown (in orange). Analysis of the OF texts shows that they are more likely to rest and have dinner in Disneytown. For example, an OF tourist posted, “I have to say, it is a good place to rest and eat jam”.

## Discussion

This study aims to contribute to the discussion of the visualization of demographic groups’ emotions and the emotional differences between demographic groups in theme parks. This discussion focused on three aspects: (1) the advantages of our visualization, (2) the variations across groups’ emotions, and (3) the application of our findings.

Facial expressions and circumplex models of affect were combined to visualize the emotion distribution of each demographic group. Previous studies have quantified one or two positive emotion dimensions, such as pleasure and happiness. However, tourism experiences not only give rise to tourists’ positive emotions but also lead to negative emotions. Compared with positive emotions, negative emotions require more research attention. Investigating the negative emotions of tourists is the key to improving the tourist experience. The proposed emotion distribution graph can clearly visualize both positive and negative emotions in a theme park. The second advantage of our visualization method is that emotional differences between scenic spots can be presented effectively. The circumplex model of affect can classify many emotion dimensions; based on this model, our graph does not ignore any slight emotional differences. The third advantage is that our study provides insightful information about the complex tourists’ emotions of each demographic group. Existing studies have focused mainly on tourists as a whole and have focused on exploring patterns of emotional dynamics. However, what are the differences between tourist groups with different demographic characteristics? In this study, facial expressions were used to quantify emotion, age and gender. With the support of facial expression data, our method has the ability to visualize the emotion distribution of each demographic group.

In “Emotion distribution analysis” section, we found that there are unique characteristics of emotion distribution for each group. By combining various analysis methods, the emotion distribution can be generally evaluated, and the structure of the distribution can be detected. For example, the ellipse of the TMs demonstrates that their emotion distribution has the highest central trend and the largest area. TMs have no cluster because of the loose structure of emotion points in the graph. Interestingly, the motivation effects of theme lands on valence and arousal are not the same for some groups. Owing to the design objects of theme lands, some themes cannot significantly influence both the valence and arousal of some groups. For example, Adventure Isle was designed to increase the degree of excitement among tourists. In the case of the AFs and OMs, Adventure Isle evokes the greatest arousal, but this theme land does not give rise to the highest valence.

In “Emotion distribution analysis” section, the results demonstrate that there is considerable variation between the demographic groups’ emotions. This is not surprising, as the effects of demographic characteristics on tourists’ emotions have been discussed in recent years. A striking finding is that the TFs are more interested in Treasure Cove and Adventure Isle than the TMs. These two theme lands were designed with some adventure activities. A possible explanation is that TFs are more easily attracted by adventure activities than TMs. One unanticipated result is that both the AMs and OMs showed less excitement in Disneytown than the TMs. The TMs are interested in the food in Disneytown. In China, most adult and older males took on a large part of the responsibility of earning money for their families. They know how hard it is to make money. Therefore, AMs and OMs are more sensitive to the price of goods. The AMs and OMs complained about the high prices and posted about their negative emotions on the social media platform. However, most TMs have fewer burdens when they consume using their parents’ money. The most interesting finding is that the OFs do not feel as much pleasure or excitement in the Gardens of Imagination as the TFs and AFs. The OFs have a clear negative attitude towards the Gardens of Imagination. Based on the posted texts, we find that the romantic factors in the Gardens of Imagination have difficulty attracting the OMs.

**Figure 6:**
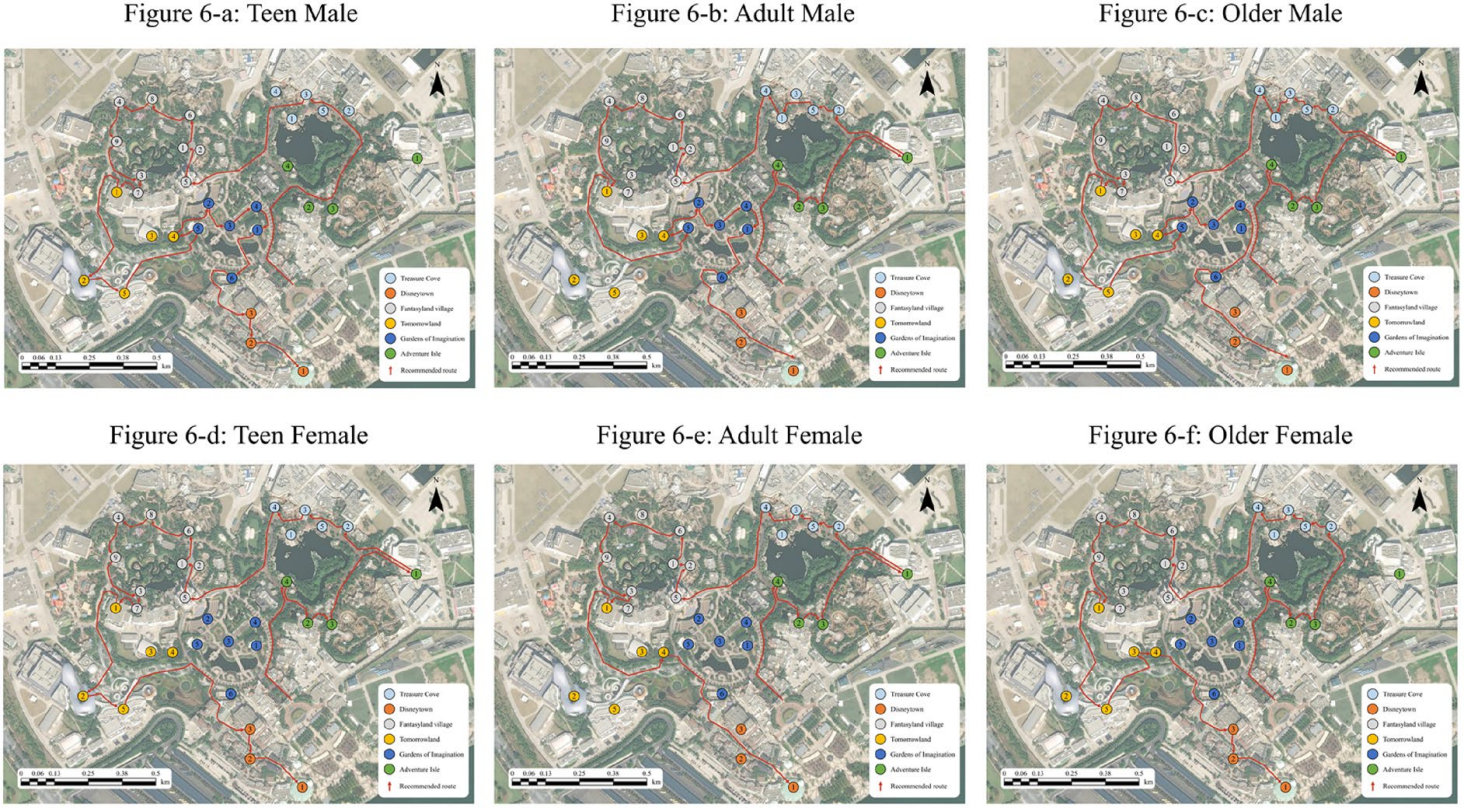
The optimized routes for each group. The optimized routes, connected with scenic spots, prevent each demographic group from having bad emotional experiences. Figures were created using ArcGIS 10.8.2 (https://www.esri.com/en-us/home).

The results of this study have significant practical implications. Our findings provide support for designing optimized travel routes for different demographic groups. For each group, we selected scenic spots that evoke both positive valence and high arousal. The scenic spots in the first quadrant for each group are shown in Table 6. By considering the constraints of the road network within the theme park, the selected scenic spots were connected with each other. The optimized routes for each demographic group were generated and are shown in Fig. 6. Our optimized route was designed based on the emotion distribution characteristics of each group and can significantly improve the tourist experience and the degree of satisfaction. These routes have obvious differences from each other. Our routes were built on the basis of emotional characteristics without considering tourist flow. Tourist flow changes dramatically and can result in crowds and long waiting lines during holidays. The congestion of tourist flow within a theme park can influence tourists’ experiences. It is necessary for managers to monitor tourist flows and then update routes.

**Table 6:** The scenic spots in the first quadrant for each group.

Groups	Theme name	Scenic spot name	Groups	Theme name	Scenic spot name
TM	Treasure Cove	The Snackin’ Kraken	TF	Treasure Cove	Eye of the Storm: Captain Jack’s Stunt Spectacular
	Disneytown	World of Disney Store			The Snackin’ Kraken
		The Dining Room			Pirates of the Caribbean Battle for the Sunken Treasure
		The Cheesecake Factory		Disneytown	World of Disney Store
	Fantasyland village	Alice in Wonderland Maze			The Dining Room
		Voyage to the Crystal Grotto			The Cheesecake Factory
		Tangled Tree Tavern		Fantasyland village	Alice in Wonderland Maze
		“Once Upon a Time” Adventure			Voyage to the Crystal Grotto
		Seven Dwarfs Mine Train			Tangled Tree Tavern
		Be Our Guest Boutique			“Once Upon a Time” Adventure
		Hunny Pot Spin			Seven Dwarfs Mine Train
		Traveling Troubadours			Be Our Guest Boutique
	Tomorrowland	Peter Pan’s Flight			Hunny Pot Spin
		TRON Lightcycle Power Run			Traveling Troubadours
		Marvel Universe		Tomorrowland	Peter Pan’s Flight
		Selfie Spot with Mickey			TRON Lightcycle Power Run
		Intergalactic Imports			Intergalactic Imports
	Gardens of Imagination	Fantasia Carousel		Adventure Isle	Soaring Over the Horizon
		Golden Fairytale Fanfare			A Celebration of Duffy and Friends at Tribal Table
		Gardens of Imagination			Roaring Rapids
		Garden of the Twelve Friends			Adventure Isle
		Dumbo the Flying Elephant			Explorer Canoes
		Mickey’s Storybook Adventure			
AM	Treasure Cove	Shipwreck Shore	AF	Treasure Cove	Eye of the Storm: Captain Jack’s Stunt Spectacular
		Eye of the Storm: Captain Jack’s Stunt Spectacular			The Snackin’ Kraken
		Pirates of the Caribbean Battle for the Sunken Treasure			Pirates of the Caribbean Battle for the Sunken Treasure
		Tortuga Treats			Tortuga Treats
	Fantasyland village	Alice in Wonderland Maze		Disneytown	World of Disney Store
		Mad Hatter Tea Cups		Fantasyland village	Alice in Wonderland Maze
		Tangled Tree Tavern			Mad Hatter Tea Cups
		“Once Upon a Time” Adventure			Voyage to the Crystal Grotto
		Seven Dwarfs Mine Train			Tangled Tree Tavern
		Hunny Pot Spin			“Once Upon a Time” Adventure
	Tomorrowland	Peter Pan’s Flight			Seven Dwarfs Mine Train
		Selfie Spot with Mickey			Traveling Troubadours
	Gardens of Imagination	Fantasia Carousel		Tomorrowland	Peter Pan’s Flight
		Golden Fairytale Fanfare			Selfie Spot with Mickey
		Gardens of Imagination			
		Garden of the Twelve Friends		Adventure Isle	Soaring Over the Horizon
		Dumbo the Flying Elephant			A Celebration of Duffy and Friends at Tribal Table
		Mickey’s Storybook Adventure			Roaring Rapids
	Adventure Isle	Soaring Over the Horizon			Adventure Isle
		A Celebration of Duffy and Friends at Tribal Table			Explorer Canoes
		Roaring Rapids			
		Adventure Isle			
		Explorer Canoes			
OM	Treasure Cove	Shipwreck Shore	OF	Treasure Cove	Eye of the Storm: Captain Jack’s Stunt Spectacular
		Eye of the Storm: Captain Jack’s Stunt Spectacular			The Snackin’ Kraken
		The Snackin’ Kraken			Pirates of the Caribbean Battle for the Sunken Treasure
		Pirates of the Caribbean Battle for the Sunken Treasure			Tortuga Treats
		Tortuga Treats		Disneytown	World of Disney Store
	Fantasyland village	Voyage to the Crystal Grotto			The Dining Room
		Tangled Tree Tavern			The Cheesecake Factory
		“Once Upon a Time” Adventure		Fantasyland village	Alice in Wonderland Maze
		Be Our Guest Boutique		Tomorrowland	Tangled Tree Tavern
		Hunny Pot Spin		Gardens of Imagination	Seven Dwarfs Mine Train
	Tomorrowland	Peter Pan’s Flight		Adventure Isle	Hunny Pot Spin
		Selfie Spot with Mickey			Traveling Troubadours
		Intergalactic Imports		Tomorrowland	Peter Pan’s Flight
	Gardens of Imagination	Fantasia Carousel			Marvel Universe
		Golden Fairytale Fanfare			Selfie Spot with Mickey
		Gardens of Imagination			Intergalactic Imports
		Garden of the Twelve Friends		Adventure Isle	A Celebration of Duffy and Friends at Tribal Table
		Dumbo the Flying Elephant			Roaring Rapids
		Mickey’s Storybook Adventure			Adventure Isle
	Adventure Isle	Soaring Over the Horizon			Explorer Canoes
		A Celebration of Duffy and Friends at Tribal Table			
		Adventure Isle			
		Explorer Canoes

The limitations of this study need to be noted. Our study is driven by online facial expression data. Some publishers may underexaggerate or overexaggerate their emotions on social media platforms. There may be bias in the emotions extracted from online facial expressions^84,113^. People-related images mainly reflect the tourism emotions of publishers; some of these emotions may be distorted by the publishers’ subjective consciousness and may not influenced by the scene of the theme park. In future work, we will focus on recognizing the distorted facial expressions and correcting the tourists’ emotions extracted from online images.

## Conclusions

Since this study attempted to visualize both positive and negative emotions in each demographic group, it provides quantitative evidence supporting these striking and interesting findings. The method framework can be the basis of further research in this area to explore the differences in emotions across diverse groups. Furthermore, the findings can help managers of theme parks reconsider marketing policies to provide tourists with satisfying experiences.

To conclude, this paper tapped into a novel domain within tourism emotion research and focused on revealing the emotional characteristics of different demographic groups. The study proposed an emotion distribution graph for visualization, and then carried out a trend and variation analysis of the emotions of each group. The results indicate that the emotion distribution of each group has unique trend characteristics. Furthermore, there are unanticipated differences in emotions across demographic groups. Researchers and managers should have a deep understanding of the variation between groups when they develop new marketing plans or conduct studies related to tourists’ emotions. The old approach of ignoring tourists’ demographic attributes needs to be updated, as it can decrease the accuracy of emotion monitoring or even result in negative experiences for tourists. This paper presents a methodological framework that visualizes and analyzes groups’ emotions with varying genders and ages. The framework was applied to Disneyland, and the results indicate that demographic groups have considerably different emotional characteristics. Our method can be easily applied to other tourist destinations; however, further studies are needed to confirm whether the characteristics identified in our study are cross-cultural and cross-national.

## Data Availability

The datasets generated during the current study are available from the corresponding author upon reasonable request. The data are available from Sina Weibo, but restrictions apply to the availability of these data, which were used under license for the current study and are not publicly available. The related codes in our study have been shared on GitHub. The website is https://github.com/wuhaozede/theme-park-emotion-visualization.
